# Is Prophylaxis the Only Way Out for Cytokine Release Syndrome Associated With Chimeric Antigen T-cell Therapy?

**DOI:** 10.7759/cureus.17709

**Published:** 2021-09-04

**Authors:** Prashil Dave, Elisa Pallares Vela, Ivan Cancarevic

**Affiliations:** 1 General Medicine, California Institute of Behavioral Neurosciences & Psychology, Fairfield, USA; 2 Internal Medicine, California Institute of Behavioral Neurosciences & Psychology, Fairfield, USA

**Keywords:** cytokine release syndrome (crs), chimeric antigen receptor t-cell therapy, cancer-immunotherapy, management strategies, leukaemia, oncology clinical trials, relapsing multiple myeloma, incidence rate

## Abstract

Chimeric antigen receptor T-cell (CAR-T) therapy is a new advancement in hematology and oncology with its use in treating many refractory malignancies. Cytokine release syndrome (CRS) is CAR-T's clinically hazardous side effect, ranging from mild to life-threatening events. It was one of the first side effects detected with CAR-T. We conducted a literature review using PubMed (MeSH) to study CRS incidence after the administration of CAR-T to reflect its clinical importance. Nine studies are mentioned, with a total of 1357 patients enrolled for different types of refractory/relapsed cancers, and an average incidence of CRS of 64% is being noted. We have also stated numerous studies which mentioned the use and effectiveness of the commonly used drugs like tocilizumab, corticosteroids, and some new drugs. Although statistical data on CRS's conservative and supportive management is not available, the role of different supportive measures is evident. An overview of how it sets the framework of a peri-management approach has been considered. Through heightened incidence and relatively complex management of CRS, we would like to raise the question of the need for early prophylaxis against CRS when considering CAR-T. The need for more clinical trials in the future to prove the effectiveness of the latter is stated.

## Introduction and background

Chimeric Antigen Receptor (CAR) Therapy is T-cell immunotherapy used to treat lymphoid malignancies [1]. Its use began with treating pediatric acute lymphoblastic leukemia (ALL) and diffuse large B-cell lymphoma (DLBCL) [1]. Prolific advances are being made to make it proficient in treating adult refractory Hodgkin's lymphoma, acute myeloid leukemia (AML), multiple myeloma (MM), and neuroblastoma [2]. It comprises genetically engineered CAR-T cells designed to target tumor cell antigens and spare the healthy cells [3]. The introduction of these cells into the patient results in extensive but controlled stimulation of cell multiplication, rendering them active and cytotoxic to the tumor cells [4]. This cytotoxicity results from stimulation factors such as growth factors, cytokines, and interleukins; many of these form the basis of the mechanism of the common side effects seen with this therapy [4].

The introduction of chimeric antigen receptor T-cell (CAR-T) therapy was acknowledged by many as a new ray of hope in the field of oncology [5]. However, some of its side effects have fatal consequences [5]. The more hazardous of such toxicities are cytokine release syndrome (CRS) and immune effector cell-associated neurotoxicity (ICANS) [6]. In addition, during the long course of treatment, several toxicities such as the increased risk of opportunistic infections, cytopenias, and bone marrow aplasia have also been noted [6].

Understanding the pathophysiology behind the side effects helps better understand the treatment and its outcomes [2]. Hierarchical systems have been used to assess the severity of these side effects, which heavily rely on the levels of interleukin (IL)-6, IL-8, IL-10, and others [6]. However, despite this knowledge, managing the patients appropriately in a way that does not affect their treatment outcomes is quite a challenge [6]. Several clinical trials have been done regarding efficacy and safety; the results of three such are: ZUMA-1, JULIET, and TRANSCEND have been quite insightful [6]. According to the ZUMA-1 trial, the incidence of CRS as a mean of most clinical trials was 70% in ALL patients and 94% in non-Hodgkin's lymphoma (NHL) patients [6]. The incidence of neurotoxicity ranged from 13% to 64% in ALL and 7% to 31% in NHL patients [6].

Cytokines are protein molecules that act as either pro-inflammatory or anti-inflammatory immunomodulators [7]. Both work in conjunction as immunoregulators of each other and are responsible for their physiologic and pathologic effects [7]. The goal of these regulators is to heighten the immune response to fight against pathogens and tumor cells [7]. A unique example is interferon (IFN)-beta; it reduces the immune response and protects many inflammatory disease states, such as multiple sclerosis [8]. Another important cytokine, IL-6, acts via signal transduction and Janus kinase (JAK)/signal transducer and activator of transcription (STAT) pathway modulation; it is an acute phase response reactor to pathological infection and inflammatory states [9]. On one end of the spectrum is the role of cytokines as important biological markers for disease states, proving to be both diagnostic and therapeutic, and on the other is their pathophysiological role in the various diseases [10]. From autoimmune to neurological disorders, an association with cytokines is well documented [10]. Typical examples are IL-6 and rheumatoid arthritis, IFN-alpha and hepatitis B, IL-10 and Crohn's disease, and many others [10]. Overstimulation of cytokines results in two common conditions: cytokine storm (CS) and CRS, which have similar clinical phenotypes with significant differences in their characteristics [11]. CS is the acute excessive activation of the cytokines independent of tumor therapy, resulting in hemodynamic instability [11]. In contrast, CRS is a systemic inflammatory response syndrome (SIRS), causing overstimulation of the immune system but with a less acute course, even presenting days or weeks after initiation of therapy [11].

Incidence of CRS with different CAR-T therapies and the knowledge of the type of treatment approach required to prevent its progression into a life-threatening stage is essential [12]. Thus, with the help of this article, we are trying to elucidate the incidence and management of CRS associated with CAR-T; to better understand one of the most common side effects associated with this CAR-T for the betterment of patient toxicity profile [12]. The concluding results eventually set the base for early consideration of prophylaxis to bring down the incidence rates [12].

Methodology

We performed an elaborated PubMed (MeSH) and Google Scholar search from the last two decades. We used the keywords 'chimeric antigen therapy', 'immunotherapy' or ‘axicabatagene’, and combined them using the Boolean operator 'and' with the words 'cytokine release syndrome,' 'toxicity,' 'incidence,' 'tocilizumab,' 'prophylaxis', and 'management.' We included studies comprising of clinical trials, randomized control trials, case reports, and systematic reviews performed in humans only. We excluded studies that did not meet these criteria. In total, we used 33 articles to write this review. The languages included were in English, with only one article being in Chinese, which was google translated.

## Review

Incidence

The rate of occurrence of CRS with CAR-T is an expanded way to understand the incidence [13]. Therefore, we focused on incidence as per the grading system. Management of CRS is a multimodality approach with integrated care through intensive care unit (ICU) staff and oncologists [13]. Grading systems help compartmentalize CRS and help focus on the respective treatment modalities [14]. The common grading systems are Lee criteria, Penn criteria, Common Terminology Criteria for Adverse Events (CTCAE) version 5.0, Memorial Sloan Kettering Cancer Center (MSKCC) criteria, and CAR toxicity (CARTOX) criteria [14,15]. 

Shah et al. conducted a single-center, phase I trial focussing on cluster of differentiation (CD)22-targeted CAR-T cells in children and young adults with relapsed hematologic malignancies [16]. A group of 64 participants, comprising children and adults between ages 3-30, were included in the trial out of which 58 received infusion [16]. The Mann-Whitney U test compared unpaired data sets, and lymphodepletion was obtained initially through fludarabine and cyclophosphamide [16]. CRS incidence was seen in 50 patients (86.2%) of any grade, using the Lee et al. [15] system for grading [16]. Grade 1-2 was seen in 45 patients and grade >=3 in five patients [16].

Curran et al. conducted another multicenter phase I trial with 19-28z (a CD28-containing, second-generation CAR) CAR-T cell therapy on age group 1-22.5 with relapsed B-ALL (B-cell acute lymphoblastic leukemia) [17]. Inclusion criteria encompassed >=13 years at diagnosis, and the study included 25 patients [17]. CRS was seen in 20 patients (80%) with grade >=3 in four (16%) participants [17]. Grading was done according to the National Cancer Institute (NCI) grading system, and cyclophosphamide was used for conditioning therapy [17].

Raje et al. conducted an open-label phase I study throughout various centers in the USA using bb2121 CAR-T therapy for relapsed/refractory multiple myeloma [18]. Clopper-Pearson 95% CI was used for statistical significance [18]. Thirty-six patients (age group 37-75) were included in the study from 2016 to 2018, and 33 received the infusion [18]. CRS was seen in 25 (76%) patients with grade 1 or 2 in 23 (70%), grade 3 in two (6%) patients, and zero cases with grade 4 [18]. Lee et al. [15] scale was considered for grading and lymphodepletion conducted with fludarabine and cyclophosphamide [18].

Wang et al. conducted a multicenter phase II ZUMA-2 trial with KTE-X19 (anti-CD19) CAR-T therapy for relapsed/refractory mantle cell lymphoma at 20 different sites throughout the USA and Europe [19]. Sixty-eight out of the total 74 patients received the infusion [19]. CRS was seen in 62 (91%) patients with grade 1 in 20 (29%), grade 2 in 32 (47%), grade 3 in eight (12%) and grade 4 in two (3%) patients [19]. Grading was done using Lee at el. [15] scale parameters, and conditioning chemotherapy was done using cyclophosphamide [19].

Ramos et al. study is an open-label, two parallel phase I/II trial for relapsed/refractory Hodgkin’s lymphoma using anti-CD30 CAR-T [20]. Forty-one patients received infusion, out of which 10 developed CRS (all of which were grade 1) [20]. CTCAE, version 4.0 scale used for grading, and lymphodepletion was conducted using fludarabine [20].

Neelapu et al. conducted a multicenter phase II trial for refractory large B-cell lymphoma, including 22 study centers from November 2015 to September 2016 [21]. One hundred eleven patients were enrolled, of which 101 received infusion (age-range 23-76 with 24 patients above 65) [21]. Seventy-eight were refractory to 2nd line therapy, and 21 had relapsed after autologous stem cell transplantation [21]. CRS occurred in 94 patients (93%) with grade 1 in 37%, grade 2 in 44%, grade 3 in 9% and grade 4 in 3% [21]. Pyrexia was the most common presenting symptom. Grading was done using the Lee et al. scale [15], and conditioning therapy was done using low-dose cyclophosphamide and fludarabine [21].

Grigor et al. conducted a systematic review and meta-analysis study with MEDLINE, Embase, and Cochrane trials [22]. It included 42 hematological cancer and 18 solid tumor studies with 913 participants in total [22]. CRS was reported in 18 hematologic studies with 594 participants and a pooled prevalence of 55.3% [95% CI, 40.3%-69.4%] patients [22]. CRS was also reported in two solid cancer studies (17 patients) and a pooled prevalence of 5.4% [95% CI, 0.8-30.2%] patients [22].

Schuster et al. conducted an open-label, phase II pivotal study for adult patients who had relapsed/refractory diffuse large B-cell lymphoma using tisagenlecleucel CAR-T therapy [23]. Ninety-three patients were reported to have received the infusion [23]. CRS incidence was 64 (58%) patients, with grades 1-2 seen in 40 patients, grade 3 in 15, and grade 4 in nine patients [23]. Grading was done using the Penn scale, and lymphodepletion conditioning therapy included fludarabine, cyclophosphamide, or bendamustine [23].

Management

The severity of clinical presentation of CRS increases in ascending order as per the grade [23]. Management strategies (Table 1) comprise drug therapy along with appropriate supportive care measures [23-25]. 

**Table 1:** CRS clinical presentation and management strategies BP: blood pressure

Author	Grade (Lee grading system)	Clinical presentation	Treatment
Schuster et al., Neelapu et al. and Lee et al. [23-25]	I	Non-life threatening symptoms: headache, myalgia, fever	Supportive care; drug therapy for refractory fever or fever >3 days
Schuster et al., Neelapu et al. and Lee et al. [23-25]	II	BP <90/60 mmHg requiring fluid support, hypoxia requiring low flow oxygen, grade 2 organ toxicity	Supportive care if no comorbidities; consider drug therapy if comorbidities
Schuster et al., Neelapu et al. and Lee et al. [23-25]	III	Amplified grade 2 symptoms: hypotension and hypoxia non-responsive to routine treatment that requires vasopressors and high flow oxygen respectively; grade 3 organ toxicity	Drug therapy with ongoing advanced supportive care
Schuster et al., Neelapu et al. and Lee et al. [23-25]	IV	Amplified grade 3 symptoms: hypotension requires multiple vasopressors and hypoxia requiring mechanical ventilation; grade 4 organ toxicity	Same as grade 3, consider mechanical ventilation

a. Corticosteroids

Turtle et al. studied 37 patients with relapsed/refractory NHL (age group 22-70), out of which 32 received infusion [26]. Twenty patients reported CRS, and four received corticosteroids with consequent complete resolution [26]. Kaplan-Meier method was used for time-to-event analysis and reverse Kaplan-Meier for median follow-up time [26].

Liu et al. depicted a study of 68 patients with relapsed/refractory B-ALL, where 64 developed CRS: Grade 1 in 10 patients, grade 2 in 44 patients, and grade 3 in 10 patients [27]. Dexamethasone and/or methylprednisone were administered to 42 patients (30 with grade 2 CRS and 10 with grade 3) [27]. Twenty-three out of 42 patients received high-dose steroids, and the impact of steroids was evaluated, which showed 40 patients with complete remission and two with partial remission [27].

Teachey et al. studied 51 patients (ages 5-72) in a two cohort (pediatric and adult) evaluation for relapsed/refractory ALL (R/R ALL) [28]. Incidence of CRS was 48 (94%) patients [28]. Grade 1-2 is seen in 18 patients, grade 3 in 16 patients, and grade 4-5 in 14 patients [28]. Fisher’s and Wilcoxon rank-sum methods were used for group comparisons [28]. Out of those affected, 12 patients were treated with corticosteroids and reported clinical improvement [28].

In all the studies, doses varied according to the patient profile. Points considered were age, BMI, grade of CRS, and clinical status [24]. Lee et al. recommend using a standard corticosteroid (e.g., methylprednisolone) dose of 2 mg/kg/day with gradual weaning [25]. Consideration to adding dexamethasone 10 mg IV every six hour for refractory hypotension (increase to 20 mg IV if refractory) and grade 2 or more hypoxia is mentioned [25]. Alternatively, methylprednisolone can be used with a dose of 1 g/day IV for grade 4 with an addition of dexamethasone 0.5 mg/kg is recommended for patients with concomitant neurological symptoms [23-25].

b. Tocilizumab

Kadauke et al. studied an open-label 2-cohort pilot prospective clinical trial conducted at the University of Pennsylvania for relapsed/refractory B-ALL [29]. It included 80 participants, ages 1-24 years (70 infused with CAR-T treatment), and allocation was non-randomized [29]. Fifteen patients with a high tumor burden were given tocilizumab pre-emptive treatment upon the onset of symptoms like a high-grade fever [29]. The occurrence of grade 4 CRS was 27% (95% CI, 8 to 55) without affecting CAR-T efficiency, suggesting an expected decrease in progression to severe CRS with correctional use of tocilizumab [29]. Historical cohort without pre-emptive use of tocilizumab was considered for comparison, in whom severe CRS developed in 50% of the patients, suggesting a decline of 23% between those given pre-emptive tocilizumab versus those who weren’t [29].

For the treatment of severe CRS (sCRS), Davila et al. reported 16 adults with refractory or relapsed B-ALL treated with CAR-T in a phase I trial using Spearman rank-order correlations (p>=0.4) [30]. Six adults developed sCRS, out of which three were treated with tocilizumab [30]. Resolution of symptoms with no relapse was noted [30]. Furthermore, no reduction in expansion of CAR-T cells was reported [30].

JULIET TRIAL: Schuzter et al. studied the response of tocilizumab in patients in the JULIET trial, which included 111 patients with relapsed /refractory diffuse large B-cell lymphoma [23]. Sixty-three patients had CRS according to the Lee scale, with 45 patients having grade 0-2 (out of which two received tocilizumab) and 19 patients had grade 3-4 CRS (out of which 14 received tocilizumab) [23]. Six patients required one dose, ten patients required two doses, and consequent resolution of symptoms was reported [23].

CARTOX working group: Neelapu et al. reported using tocilizumab to represent the recommendation as incurred from the study of CARTOX, which involved more than 100 adults [24]. They presented a clinical case study to explain the same [24]. Grading was done using the Lee scale, and tocilizumab use was deemed effective from grade 2 and above [24].

ZUMA-1 trial: Locke et al. studied the ZUMA-1 trial, a multicenter trial at 22 sites in the USA and Israel for patients >18 years of age [31]. The type of malignancy the study focused on was large B-cell lymphoma and subtypes like DLBCL, primary mediastinal B-cell lymphoma, and transformed follicular lymphoma [31]. One hundred nineteen patients were enrolled, out of which 108 received CAR-T therapy [31]. Forty-nine patients who received tocilizumab (30 patients one dose, 13 patients two doses, and two patients three doses) resolution of symptoms with no reduction in expansion of CAR-T cells [31].

In the Teachey et al. study mentioned before for R/R ALL, out of 48 who developed CRS; seven subjects had grade 3, and 14 had grade 4-5 [28]. All of them were administered tocilizumab [28]. Gradual follow-up on the cytokines revealed a marked decrease in their levels and resolution of clinical symptoms [28].

Le et al. studied a total of nine clinical trial studies of CAR-T [32]. They conducted a retrospective analysis to state the efficacy of tocilizumab in patients who developed CRS in those studies [32]. A total of 60 patients were treated with tocilizumab within age groups 2-12 (21 patients), 13-17 (15 patients) and >=18 (24 patients) [32]. It included ALL (47), DLBCL (12), and primary mediastinal B-cell lymphoma (PMBCL) (1) malignancies [32]. They noted 53-69% efficacy results after assessing the response of days two, seven, and 21 and showed that most patients responded in the first seven days [32].

In all the studies, doses varied according to the patient profile. Points considered were age, BMI, grade of CRS, and clinical status [24]. Reliable studies conducted by Lee et al. recommend administering tocilizumab over one hour at a dose of 4-8 mg/kg [25]. If there is no significant clinical improvement, repeat the dose after 24-48 hrs [25]. 

c. Vasopressors

Due to the scarcity of data available for pressors, study results are limited to case reports. In a case report published by Lee et al., an 11-year-old female patient with relapsed B-ALL post-hematopoietic stem cell transplantation (HSCT) was treated with CAR-T cell therapy, with subsequent lymphodepletion done with cyclophosphamide and fludarabine [25]. Her CRS symptoms began on the fourth day and were started on pressor drugs dopamine and norepinephrine [25]. Her symptoms lasted for 10 days and continued pressor support was given throughout [25]. Symptoms finally receded by day 13, and the patient was discharged [25]. It is suggested to consider using high-dose vasopressors: Norepinephrine (>= 20 microgram/kg/min), dopamine (>=10 microgram/kg/min) or phenylephrine (>=200 microgram/kg/min) for grade 3 and higher [25]. If the patient is refractory to intravenous fluids and tocilizumab, start vasopressors [24,25].

A detailed overview of different studies on management (Table 2) with an emphasis on the type of remission (complete or partial) suggests the scientific breakthrough achieved by certain drugs widening the horizon for more clinical advances [25]. 

**Table 2:** CRS-related treatment medications CR: complete resolution; PR: partial resolution [of symptoms]; DLBCL: diffuse large B-cell lymphoma; B-ALL: B-cell acute lymphoblastic leukemia; NE: Norepinephrine; R/R: relapsing-remitting; HSCT: hematopoietic stem cell transplantation; NHL: non-Hodgkin lymphoma; ALL: acute lymphoblastic leukemia; R/R MM: relapsed/refractory multiple myeloma

Study	Type of malignancy	No. of patients in the study	Incidence of CRS	Drug used	No. of patients receiving the drug	Result
Schuster et al. [23]	R/R DLBCL	111	63	Tocilizumab	16	CR
Lee et al. [25]	Relapsed B-ALL post-HSCT	Case report	Case report	Vasopressors (dopamine and NE)	Case report	CR
Turtle et al. [26]	R/R NHL	37	20	corticosteroids	4	CR
Liu et al. [27]	R/R B-ALL	68	64	corticosteroids	42	40 CR and 2 PR
Teachey et al. [28]	R/R ALL	51	48	corticosteroids	12	CR
Teachey et al. [28]	R/R ALL	51	48	Tocilizumab	21	CR
Kadauke et al. [29]	R/R B-ALL	80	9 (severe CRS only)	Tocilizumab (pre-emptive only)	15	The incidence of severe CRS was 23% less in those receiving pre-emptive tocilizumab than those who did not.
Davila et al. [30]	R/R B-ALL	16	6 (severe CRS only)	Tocilizumab	3	CR
Locke et al. [31]	Large B-cell lymphoma	119	108	Tocilizumab	49	CR
Zhang et al. [33]	R/R MM	8	8	Etanercept	3	CR

Other medications

Etanercept

Zhang et al. were the first to study the effects of etanercept (TNF-alpha inhibitor) in multiple patients (age group 53-67) [33]. They enrolled eight patients with relapsed/refractory multiple myeloma in their study in a period of three years (March 2017 to March 2020) [32]. All of them developed CRS, and out of them, three grew significantly elevated levels of TNF-alpha [33]. Etanercept (25 mg) was used for patient one, 50 mg was used for patient two after failing to control the symptoms after tocilizumab use, and 25 mg was used for patient three on day 17 pre-empted due to re-emergence of CRS [33]. All of these patients reported resolution of symptoms [33].

Infliximab, Anakinra, and Siltuximab

Studies by Lee et al. show the use of anakinra and infliximab, IL-1 R inhibitor and anti-TNF-alpha antibodies, respectively, in CRS due to other causes [25]. Siltuximab is a monoclonal antibody against IL-6, and Riegler et al. have recommended considering its use if a patient is refractory to tocilizumab and corticosteroids [14].

Supportive care

There are numerous supportive measures, and conducting a clinical study on any of them individually is beyond the scope of a clinical researcher. Therefore, we have mentioned the special efforts opted after reviewing the studies cumulatively.

Even though medications have always been an attractive choice in treating CRS, supportive care holds an integral role in the overall management [25]. Starting from grade 1 to grade 4, continuous supportive care in the form of O_2_ support (when hypoxia <40% for grade 2 and >40% for grade 3 and above), maintenance of IV fluids (depends on the blood pressure), antibiotics in patients who are neutropenic and mechanical ventilation if required (usually grade 4 or worse) is inevitable [23-25]. Antipyretics (e.g., paracetamol) and analgesics are frequently used as well [24,25]. Considerations for doing a chest X-ray as early as grade 1 are suggested and transfer to ICU is recommended upon grade 3 and more [25]. Close monitoring of organ function, with an echocardiogram for cardiac status, is also reflected in most studies [23-25].

Limitations

A thorough review of additional articles was impossible since we did not get access to complete papers and hence, limit the search. Also, many of the trials were found to be in phase I and will take several more years to complete. Therefore, additional information, such as results for later phases of the trial, was not derived for such articles.

## Conclusions

The collaboration between CAR-T and clinical improvement has been proven, but special consideration to CRS, its grade, frequency, and management is necessary. This review concludes that CRS incidence is much higher than anticipated, with a frequency falling anywhere between 55-93%. Although primarily in the initial phase of the study, sufficient data is extracted throughout the literature for standardized treatment like tocilizumab and corticosteroids. With many clinical trials concluding soon, there is an established hope that advances are upcoming for comprehensive CRS management. The need for scientific research and pharmacological innovations is inevitable, and we hope that in the future, there will be numerous clinical trials that would take place. With such high-level incidence, a complex peri-management profile, the inevitable question of consideration of prophylaxis against CRS concurrent to CAR-T administration is put forward. We hope there are clinical studies conducted on the same subject in the future. This will help not eradicate but to lessen the occurrence of CRS and its significant impact on patient profile. 
